# Experimental Design as a Tool for Optimizing and Predicting the Nanofiltration Performance by Treating Antibiotic-Containing Wastewater

**DOI:** 10.3390/membranes10070156

**Published:** 2020-07-19

**Authors:** Dalva Inês de Souza, Alexandre Giacobbo, Eduardo da Silva Fernandes, Marco Antônio Siqueira Rodrigues, Maria Norberta de Pinho, Andréa Moura Bernardes

**Affiliations:** 1 Post-Graduation Programme in Mining, Metallurgical and Materials Engineering, (PPGE3M), Federal University of Rio Grande do Sul (UFRGS), Av. Bento Gonçalves, n. 9500, Agronomia-Porto Alegre-RS, CEP 91509–900, Brazil; dalvines@gmail.com (D.I.d.S.); amb@ufrgs.br (A.M.B.); 2 Post-Graduation Programme in Production Engineering, Federal University of Rio Grande do Sul (UFRGS), Av. Osvaldo Aranha, n. 99, Bom Fim-Porto Alegre-RS, CEP 90035–190, Brazil; edu.silva.fernandes@gmail.com; 3 Post-Graduation Programme in Materials Technology and Industrial Processes, Pure Sciences and Technology Institute, Feevale University, Rodovia RS-239, n. 2755, Vila Nova-Novo Hamburgo-RS, CEP 93525–075, Brazil; marcoR@feevale.br; 4 Chemical Engineering Department, Instituto Superior Técnico, University of Lisbon, Av. Rovisco Pais, n. 1, 1049–001 Lisbon, Portugal; marianpinho@tecnico.ulisboa.pt; 5 Centre of Physics and Engineering of Advanced Materials, CeFEMA, Instituto Superior Técnico, University of Lisbon, Av. Rovisco Pais, n. 1, 1049–001 Lisbon, Portugal

**Keywords:** nanofiltration, antibiotic in wastewater, norfloxacin, sulfamethoxazole, experimental design, factorial design

## Abstract

In recent years, there has been an increase in studies regarding nanofiltration-based processes for removing antibiotics and other pharmaceutical compounds from water and wastewater. In this work, a 2^k^ factorial design with five control factors (antibiotic molecular weight and concentration, nanofiltration (NF) membrane, feed flow rate, and transmembrane pressure) was employed to optimize the NF performance on the treatment of antibiotic-containing wastewater. The resulting multiple linear regression model was used to predict the antibiotic rejections and permeate fluxes. Additional experiments, using the same membranes and the same antibiotics, but under different conditions of transmembrane pressure, feed flow rate, and antibiotic concentration regarding the 2^k^ factorial design were carried out to validate the model developed. The model was also evaluated as a tertiary treatment of urban wastewater for removing sulfamethoxazole and norfloxacin. Considering all the conditions investigated, the tightest membrane (NF97) showed higher antibiotics rejection (>97%) and lower permeate fluxes. On the contrary, the loose NF270 membrane presented lower rejections to sulfamethoxazole, the smallest antibiotic, varying from 65% to 97%, and permeate fluxes that were about three-fold higher than the NF97 membrane. The good agreement between predicted and experimental values (*R*^2^ > 0.97) makes the model developed in the present work a tool to predict the NF performance when treating antibiotic-containing wastewater.

## 1. Introduction

Antibiotics are a class of pharmaceutical compounds with a huge input into the environment, a fact that is associated with their high consumption. In 2015, the consumption of antibiotics worldwide was estimated at 42 billion defined daily doses (DDDs) and, if consumption patterns do not change, an increase of 200% is projected for 2030 [1]. 

In fact, a great concern regarding antibiotics is that after administration, it is only partially absorbed by the patient, being the rest excreted in the urine or feces [2], reaching the urban sewage network and then the urban wastewater treatment plants (UWWTPs). In turn, these have conventional treatment processes, which are inefficient in removing antibiotics and other micropollutants [2,3,4]; thus, the discharged wastewater ends up contaminating the different ecosystems [5]. For this reason, UWWTPs are considered as one of the main sources of antibiotic release into the environment [6], discharging them in concentrations ranging from ng L^−1^ to µg L^−1^ [7,8,9,10]. Likewise, wastewater from the pharmaceutical industry is another important source of contamination, generally containing pharmaceuticals concentration in the range of mg L^−1^ [11,12], but that may reach a magnitude of g L^−1^ [13]. In a survey on the occurrence of antibiotics in wastewater treatment plants, Wang et al. [14] reported sulfamethoxazole (SMX), clarithromycin, amoxicillin, ciprofloxacin, ofloxacin, and norfloxacin (NOR) as the six antibiotics found in higher concentrations. In another study, performed by Montagner et al. [15], 708 samples of drinking, surface, and ground water and wastewater from São Paulo State (Brazil) were analyzed, and the results showed that amoxicillin, trimethoprim, cephalexin, ciprofloxacin, SMX, NOR, and ampicillin were the drugs detected with higher frequency. 

The key point of the environmental discharge of antibiotics is that the increasing exposure of microorganisms outside of human bodies contributes toward the recruitment and spread of antibiotic resistance genes among human pathogens [16], hampering the treatment of diseases caused by these pathogens. Therefore, the investigation of other technologies for removing antibiotics from wastewater is crucial. In this respect, membrane processes, such as nanofiltration (NF) and reverse osmosis (RO), are promising technologies to address this matter, increasing the safety of wastewater treatment plants. Thus, considering the different mechanisms acting in the separation, e.g., adsorption, steric hindrance, and electrostatic effects [17], and also the fact that the membranes have a molecular weight cut-off (MWCO) in the same range as the molecular weight (MW) of antibiotics [18], NF has gained prominence in the treatment of antibiotics-containing wastewater. 

The general idea about predicting NF performance by designing models is related to achieving project advantages such as reducing the number of experiments for studies to scaling up NF operations, minimizing the analytical characterization processes, which are often costly, and also optimizing processes for producing reuse water. Models involving different approaches have been studied to predict the rejection of trace organic compounds, including antibiotics, in NF operations.

Kim et al. [19] developed a membrane transport model to determine the diffusive and convective contributions to solute transport and rejection. The authors studied the nanofiltration of disinfection by-products (DBPs). The modeling was carried out according to a non-equilibrium thermodynamic transport equation and was able to simulate the main mechanisms of solute transport, but was not designed to predict the solute rejection coefficient.

Kong et al. [20] applied the Donnan steric pore model (DSPM) to evaluate the nanofiltration of solutions containing neutral disinfection by-products. The authors determined that the experimental rejection ratios for all the DBPs were much lower than the values predicted by the model. The DSPM is related to steric hindrance, and adsorption may have an important role on the rejection parameters. Therefore, it is important to have a combination between steric hindrance and other solute–membrane interactions. By a modified hydrodynamic model, the authors were able to evaluate the role of MW and solubility on the rejection of DBPs and concluded that further work should be carried out to really incorporate the solubility parameter into this predicting model.

A Donnan steric pore model with dielectric exclusion (DSPM-DE), incorporated with temperature functions, was evaluated by Xu et al. [21] on the treatment of 14 pharmaceuticals and personal care products (PPCP) by NF under different temperatures (5 to 25 °C). The authors were able to determine the feed temperature influence on the PCPP rejection. The model developed obtained, in some cases, over-predicted values, which was attributed to the limitation of evaluations based on steric hindrance effect, not considering the interaction between the micropollutants and membrane, as adsorption phenomena. 

A quantitative structure–activity relationship (QSAR) model was used for predicting the rejection of contaminants of emerging concern by NF. The work resulted in a general QSAR equation by using principal component analysis, partial least square regression, and multiple linear regressions. An experimental database with 106 rejection cases of contaminants of emerging concern by NF membranes as result of eight experiments was used to produce the QSAR model. After that, rejection predictions were determined for external experimental databases by using the QSAR model. Real rejections were compared to predicted ones, and acceptable determination coefficients (*R^2^*) were found (0.75 and 0.84) [22]. These developments were relative to synthetic solutions [19,20,21,22]. On the other side, Flyborg et al. [23] developed a Partial Least Squares Projection of Latent Structures (PLS) model for predicting the rejection of pharmaceutical residuals by NF using a real treated urban wastewater as feed solution. The study was carried out with the compounds found on the treated wastewater, as atenolol, azithromycin, erythromycin, diazepam, SMX, and trimethoprim, among others. The model proved to be able to predict rejection, and most of the compounds were within the 95% prediction interval, but the model coefficients need to be determined for each individual UWWTP or wastewater reuse facility.

In the present study, the NF performance was investigated in terms of membrane characteristics, operating conditions (feed flow rate and transmembrane pressure), antibiotic concentration, antibiotic molecular weight, and physicochemical characteristics, aiming the prediction of antibiotics rejection and permeate fluxes. Experimental and modeling research is carried out in the following situations: (1) treatment of wastewater containing two of the most used antibiotics with a concentration range found in pharmaceutical industries, and (2) treatment of an urban wastewater, collected after secondary treatment and incubated with the studied antibiotics, sulfamethoxazole (SMX) and norfloxacin (NOR).

## 2. Theory

The multiple linear regression (MLR) method is a statistical tool that uses explanatory variables, known as independent variables or control factors, to predict the outcome of a response (dependent) variable through a regression equation or model, assessing the strength of the relationship between the independent variable (control factor) and response variable. When the MLR analysis performed on a set of sample data results in regression coefficients (*b*_0_, *b*_1_, *b*_2_,…, *b*_n_), those represent the effect that independent variables have on the dependent one. These coefficients are estimated by the ordinary least squares method: one for each independent variable. Thus, for a dependent variable, *y*, and *m* independent variables, *x*_1_, *x*_2_,…, *x*_m_, the MLR model is given as

(1) $$y=b_{0}+b_{1}x_{1}+b_{2}x_{2}+\cdots +b_{n}x_{m}+e_{n}$$

where $b_{0}$ is the *y*-axis intercept or linear coefficient. It is also necessary to check the significance of each independent variable through the *p*-value. Using a significance level of 5%, a *p*-value less than 0.05 implies that there is a significant effect of the factor on the response variable with a 95% confidence interval [24]. The goodness of fit of an MLR model can be measured by calculating the determination coefficient (*R*^2^), which is defined as the square of the correlation between the observed values of the response variable, i.e., experimental results, and the values predicted by the model, and it gives the proportion of the variability of the response variable (*y*-values) for the control factors (*x*-values) [24]. 

The adjusted *R*^2^ (*R*^2^_adj_) is a modified version of *R*^2^ that considers the number of independent variables and the sample size. A large difference between *R*^2^ and *R*^2^_adj_ implies an excessive number of independent variables in the model [23].

## 3. Materials and Methods

Experiments were carried out with two flat sheet commercial membranes: (1) NF270, a polyamide membrane from DOW–Filmtec (Edina, MN, USA), with a MWCO of approximately 400 Da [25]; and (2) NF97, a polyamide membrane from Alfa Laval (Nakskov, Denmark), with a MWCO of approximately 200 Da [26]. These membranes were chosen because they have MWCO covering the MW range (200–500 Da) of most antibiotics reported in the literature [18].

Permeation experiments were performed in a Lab Unit M20 plate and frame filtration unit from Alfa Laval (Nakskov, Denmark), with a membrane surface area of 3.6 × 10^−2^ m^2^, which was thoroughly described in a previous work [27]. Firstly, the membranes were carefully cleaned with sodium hydroxide at pH 9.0 and 30 °C to remove residual chemicals. Secondly, they were compacted through the recirculation of distilled/deionized water (conductivity ≤ 2 µS cm^−1^), pressurized at 20 bar for 3 h. Then, they were characterized in terms of pure water permeability (*L*_PW_), as described by da Trindade et al. [28], at transmembrane pressures (Δ*P*) of 8 to 20 bar, keeping the temperature at 25 °C by an ultra-thermostatic bath. They were also characterized in terms of salt rejection (sodium chloride, sodium sulfate, and magnesium sulfate), at a Δ*P* of 10 bar, feed flow rate (*Q*_F_) of 480 L h^−1^, and feed solution of 2000 mg L^−1^. The apparent rejection coefficient (*R*) was defined as:

(2) $$R\;\left(\%\right)=\frac{C_{F-}C_{P}}{C_{F}}\times 100$$

where *C*_F_ is the solute concentration in the feed, and *C*_P_ is the solute concentration in the permeate. The permeate mass flux (*J*), in kg h^−1^ m^−2^, was calculated using Equation (3), where Δ*M* is the mass of permeate (kg), *A* is the effective membrane area (m^2^), and Δ*t* (h) is the permeation time.

(3) $$J=\frac{\Delta M}{A\;\Delta t}$$

Feed solutions with 5 and 25 mg L^−1^ of NOR and SMX, similar to concentrations found in wastewater from the pharmaceutical industry [12], were prepared in distilled/deionized water (conductivity less than 2 μS cm^−1^). These mass concentrations correspond to the molar concentrations of 15.7 and 78.3 mM for NOR, and 19.8 and 98.8 mM for SMX, respectively. The osmotic pressure of the feed solutions, which was calculated trough the Van’t Hoff equation [29,30], was neglected since it varied from 0.38 to 4.80 mbar. The pH of the antibiotic feed solutions was not altered, remaining at their natural pH when dissolved in distilled/deionized water: pH approximately 5.5 for SMX and 6.0 for NOR solutions. Both the antibiotics, with 99% purity, were purchased on a compounding pharmacy. Table 1 displays the physicochemical characteristics of the antibiotics studied.

The permeation experiments were conducted in full recirculation mode (where the retentate and the permeate streams are recirculated to the feed tank) to study the variation in the permeate fluxes and the apparent rejection coefficients. They were performed using 6 L of feed solution, evaluating each antibiotic separately, at a stabilization time of 30 min, after which permeate and feed samples were collected for chemical analysis. The antibiotics adsorption on the membranes was not investigated in this study, because it has already been reported in the literature that there was no adsorption of SMX (feed concentration of 0.5 mg L^−1^) in NF270 and other polyamide membranes [17], and a negligible adsorption of NOR, where only 5.8 µg L^−1^ was adsorbed on the membrane after 12 h of testing [18].

Experiments were carried out in duplicate as a 2^5^ factorial design, resulting in 64 experimental runs, to study the effects of control factors (antibiotic MW and concentration, MWCO of NF membrane, feed flow rate, and transmembrane pressure) on the response variables: permeate flux and antibiotic rejection (negatively skewed distribution). Factorial design experiments allow evaluating different factors simultaneously, their interactions concerning the response variables, the determination of the confidence level of the results, and the prediction of results through mathematical modeling [24]. The values of the control factors were adjusted first to the same scale, –1 and + 1 (Table 2), and the design of experiments was defined according to Yates’ algorithm by the software Minitab 17 (Table S1).

The experimental results were analyzed using the MLR method, which was chosen because it is possible to verify the coefficients of each control factor, representing its effect on the response variable and its significance. In addition, by using MLR, it is possible to make predictions of the values of the response variables for different values of the control factors. The coefficients of each control factor and factors’ interaction, as well as their respective significance, were calculated using Minitab software 17. The regression model was validated using measures of fitting quality and prediction of antibiotics rejection and permeate fluxes. Two sets of additional experiments were carried out in duplicate (100 experimental runs) to test the fitting quality of the model developed: (1) 96 runs under the same conditions used in 2^k^ factorial design (Table 2), but at Δ*P* of 8, 10, and 12 bar; (2) 4 runs using the same membranes, the same antibiotics but with the other variables at level (0), i.e., Δ*P* of 11 bar, feed flow rate of 665 L h^−1^, and antibiotic concentration of 15 mg L^−1^. In the regression analysis, all factors and interactions up to the third order were considered. The fourth and fifth-order interactions were brought together at the error term. 

Experiments were also conducted with a real urban wastewater to evaluate the effect of other organic compounds on the treatment. The wastewater was collected from an UWWTP with activated sludge in a city with 1,500,000 inhabitants in Southern Brazil. The wastewater was collected after the secondary treatment, and approximately 10 mg L^−1^ of each antibiotic (NOR and SMX) was added to the sample after collection. Then, the wastewater incubated with antibiotics was filtered through a 0.45 µm membrane to remove coarse solids. The NF experiments were conducted in concentration mode, being the retentate continuously recirculated to the feed tank, whilst the permeate was separately collected. These experiments were performed at a feed flow rate of 480 L h^−1^ and Δ*P* of 6 bar, until approximately 70% of water recovery was achieved. The characteristics of the secondary treated wastewater are presented in Table 3.

The pH and conductivity were measured using a Tek PHS-3B pH meter (Sao Paulo, SP, Brazil) and an AKSO 8306 conductivity meter (São Leopoldo, RS, Brazil), respectively. Dissolved organic carbon (DOC) was analyzed with a TOC-LCPH Shimadzu carbon analyzer (Kyoto, Japan) after sample filtration through a 0.45 µm membrane. Chemical oxygen demand (COD), total solids (TS), and total suspended solids (TSS) analyzes were performed according to the APHA Standard Methods [33]. An ultrahigh performance-liquid chromatography system from Thermo Fisher Scientific (Germering, Germany), equipped with a Supelco Drug Discovery C-18 column (with diameter, length, and pore size of 4.6 mm, 100 mm and 3 μm, respectively) and an UV–vis detector system, was used to determine the antibiotic concentrations in the feed and permeate samples. The detection wavelength was set at 277 nm for NOR and 280 nm for SMX. The detailed protocol of the chromatographic analyzes is reported in a previous study [34].

## 4. Results and Discussion

### 4.1. Membrane Characterisation

NF97 and NF270 membranes showed water permeability of 3.10 and 8.77 kg h^−1^ m^−2^ bar^−1^, respectively. For the NF97 membrane, the apparent rejection coefficients to sodium chloride, magnesium sulfate, and sodium sulfate are 89.9%, 94.9%, and 98.2%, respectively, and for the NF270 membrane, they are 46.2%, 83.9%, and 97.2%, respectively, being these values in line with the results found in the literature [35,36]. NF270 presented a water permeability that was about 2.8 times higher than the one of NF97, but lower salts rejection, particularly for sodium chloride, the monovalent salt. In fact, according to the literature, NF97 and NF270 are tight (MWCO ≤200 Da or sodium rejection ≥90%) [37] and loose [38] NF membranes, respectively.

### 4.2. Effect of Control Factors on Permeate Flux

Table 4 displays the solutions’ permeabilities (*L*_PS_), given by the slope of the straight line of permeate flux versus Δ*P*, as a function of antibiotic MW and concentration and feed flow rate for both membranes: NF97 (200 Da MWCO) and NF270 (400 Da MWCO). For both membranes and considering all conditions evaluated, the *L*_PS_ values were always very close to the *L*_PW_. Nevertheless, the NF97 membrane showed mean *L*_PS_ values slightly higher at the highest feed flow rate and with the lowest concentration of antibiotic, in which the effect of the feed concentration was more pronounced for SMX, especially for the lowest feed flow rate. 

For the NF270 membrane, the mean *L*_PS_ values of both antibiotic solutions were independent of the feed flow rate, but they showed a slight decrease with increasing feed concentration, in which the *L*_PS_–*L*_PW_ difference was more marked for NOR-containing solutions. This behavior is possibly associated with the relationship between the antibiotic MW and the membrane MWCO. As it can be seen in Table 4, the NF97 membrane, which has the smallest MWCO (200 Da), presented lower *L*_PS_ when treating solutions containing SMX, which in turn has the smallest MW (approximately 253 Da). On the other hand, the NF270 membrane, which has the highest MWCO (400 Da), presented lower *L*_PS_ when treating NOR-containing solutions, which has the largest MW (approximately 319 Da). These results indicate that the membranes are more prone to the incidence of phenomena such as concentration polarization and fouling by solutes with MW very close to the membranes’ MWCO.

Table S2 shows the coefficient and the *p*-value for each factor and factors’ interaction on permeate flux. From the analysis of experimental results, it is possible to observe that the greatest coefficients on permeate flux, in decreasing order, were membrane MWCO (factor *D*), transmembrane pressure (factor *E*), membrane MWCO–transmembrane pressure (*DE*), antibiotic MW–membrane MWCO (*AD*), antibiotic MW (factor *A*), antibiotic concentration (factor *C*), antibiotic MW–membrane MWCO–transmembrane pressure (*ADE*), antibiotic MW–transmembrane pressure (*AE*), antibiotic MW–feed flow rate (*AB*), antibiotic concentration–membrane MWCO (*CD*), antibiotic MW–feed flow rate–membrane MWCO (*ABD*), antibiotic concentration–transmembrane pressure (*CE*) and antibiotic concentration–membrane MWCO–transmembrane pressure (*CDE*).

As expected, factors *D* (membrane MWCO) and *E* (transmembrane pressure), as well as the interaction between these two factors (*DE*), have the greatest positive effects, as membranes having higher MWCO and higher transmembrane pressures usually result in higher permeate fluxes. On the other hand, antibiotic concentration (factor *C*) presents a negative effect (coefficient = –1.369) on the permeate flux, *p* = 0.000. Increasing the antibiotic concentration, a decrease in the permeate flux is observed, as there is a higher incidence of concentration polarization, which in more severe cases may result in membrane fouling. Lower permeate fluxes associated with the increase on solute concentration were already explained by the Spiegler–Kedem–Kachalsky (SKK) model, which states that the fluxes of solute and solvent are directly related to the chemical potential differences between the two sides of the membrane [39]. Other studies [7,34,40], using NF membranes treating pharmaceutics-containing wastewater, have also reported this behavior.

### 4.3. Effect of Control Factors on Rejection

Table 5 displays the rejections as a function of the MW and concentration of the antibiotic and of the feed flow rate for both membranes: NF97 (200 Da MWCO) and NF270 (400 Da MWCO). For both NF membranes, the rejection was practically independent of feed flow rate, transmembrane pressure, and antibiotic concentration. However, the membranes presented different rejections regarding the antibiotic MW. While the tightest membrane (NF97) presented similar rejections for both antibiotics, NOR and SMX, the membrane with the larger MWCO (NF270) presented low rejection to SMX (approximately 65%) and high rejections to NOR (approximately 95%). Similarly to what was previously reported for the permeate flux, the relationship between the solute MW and the membrane MWCO has also an important role on the solute rejection. The NF97 membrane has a MWCO (200 Da) that is lower than the MW of both antibiotics, SMX (MW approximately 253 Da) and NOR (MW approximately 319 Da), pointing out the steric hindrance as the main mechanism acting in the rejection of both antibiotics. On the other hand, the NF270 membrane has an MWCO of 400 Da, which, in turn, is higher than the MW of both antibiotics. Therefore, considering only the steric hindrance, very low rejections for SMX would be expected by using the NF270 membrane, unlike the moderate rejections observed, indicating thus the occurrence of other mechanisms, such as the Donnan exclusion effect. These membranes present surface charges, usually negatives, which interact with the charges from the solute; thus, they are associated with the size of the solute, and these electrostatic interactions can increase or decrease the rejection [41]. Solute and membranes with equal charges repel each other, increasing rejection, while opposite charges attract each other, promoting the adsorption of the solute on the membrane, favoring its transport and reducing the rejection [34]. 

**Figure 1:**
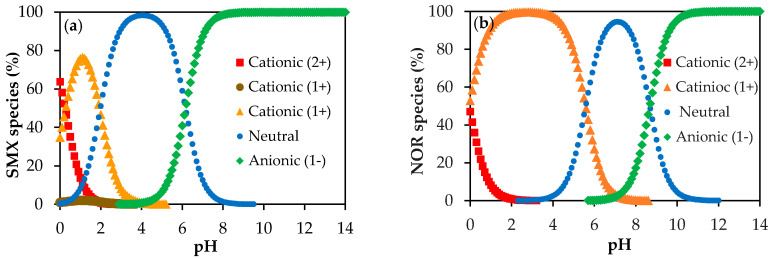
Speciation of SMX (**a**) and NOR (**b**) as a function of pH, prediction calculated data from Chemicalize software [31].

**Figure 2:**
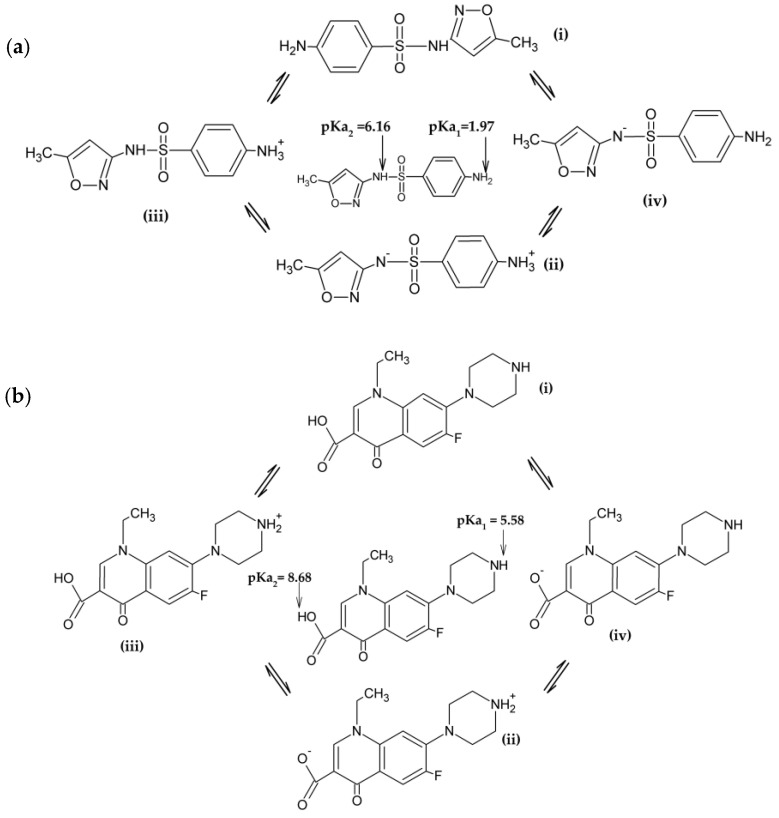
The different protonated states of SMX (**a**) and NOR (**b**) depending on pH: (i) neutral, (ii) zwitterionic, (iii) cationic, and (iv) anionic.

As reported in previous studies [34,38], polyamide membranes such as those used in this work have amphoteric characteristics and an isoelectric point in the pH range between 3.5 and 4.0, such that, in the pH range of the studied solutions (pH 5.5–6.0), they present negative surface charge. Therefore, the rejection of 65% of SMX obtained with NF270 membrane can then be attributed to the Donnan exclusion effect, once the SMX presents pKa of 1.67 and 6.16 and the SMX solutions had pH of approximately 5.5, implying that 18% of the SMX species were in the anionic form and 82% were in the neutral form (Figure 1a), while the membrane surface was negatively charged. The different protonated states of SMX depending on pH are displayed in Figure 2a. In fact, as already pointed out, the steric hindrance effect was not expected on the filtration of SMX solutions with the NF270 membrane, considering that this membrane presents a porous radius of about 0.42 nm [38] and the SMX Stokes radius is 0.39 nm (see Table 1). SMX presents also a higher diffusion coefficient.

As the surface of the NF270 membrane was negatively charged, the NOR solutions had a pH of approximately 6.0 and NOR presents pKa values of 5.58 and 8.68, while the NOR species were approximately 72.5% in the neutral form (zwitterion), approximately 27.4% in the cationic form, and 0.1% in the anionic form (Figure 1b). The different protonated states of NOR depending on pH are shown in Figure 2b. Thus, a strong attraction between the cationic species of NOR and the negatively charged functional groups from the membrane surface may occur. Therefore, the high rejection of NOR achieved with the NF270 membrane could be attributed to the steric hindrance effect and also to the adsorption of NOR on the membrane, as reported in the literature [34]. Furthermore, the NOR Stokes radius is 0.47 nm (see Table 1). 

Considering the hydrophobicity characteristics of both antibiotics (see Table 1), SMX presenting a log K_OW_ value of 0.89 has low affinity with the active polymeric layer of the membrane. NOR, with a smaller log K_OW_ (−1.03), is more hydrophilic than SMX, presenting a higher affinity with the membranes, resulting in electrostatic attraction/repulsion forces depending on pH. 

Another characteristic to be considered is the polarizability. Between the two antibiotics, SMX has the lowest values of polarizability and Stokes radius (see Table 1). Polarizability is the ability of a molecule (or atom) to have its electron cloud distorted by an external electric field, changing its original configuration. Thus, this distortion causes the molecule (or atom), originally non-polar, to acquire a dipolar moment, that is, polarizability is the ability of a molecule (or atom) to form instantaneous dipoles [23,42]. The greater the polarizability, the greater the polar character of the species, increasing the forces of interactions with other species. Longer and more elongated molecules have their electron clouds more easily distorted; that is, they are more polarizable. Conversely, small, compact, and symmetrical molecules are less polarizable, resulting in weaker electron dispersion forces. SMX is less polarizable than NOR, so the electrostatic interactions with the membrane during filtration are smaller, and a minor rejection is also expected. 

Table S3 shows the coefficients and significance of the factors and interactions on rejection. Considering the values of coefficients, they may be arranged in decreasing order of importance: *D*—membrane MWCO (−9.103), *A*—antibiotic MW (7.838), *AD*—antibiotic MW, and membrane MWCO interaction (7.683), *AE*—antibiotic MW and transmembrane pressure interaction (−0.680), and *ADE*—antibiotic MW, membrane MWCO, and transmembrane pressure interaction (−0.671), in which the first three are the most important ones. The membrane MWCO (factor *D*) has a strong negative effect (coefficient = −9.103) on rejection, when it passes from the minimum level (MWCO of 200 Da) to the maximum level (MWCO of 400 Da); that is, as the membrane MWCO increases, rejection decreases, as shown in Table 5. The antibiotic MW (factor *A*) and the interaction antibiotic MW—membrane MWCO (*AD*) have also strong effects (coefficients = 7.838 and 7.683, respectively) on rejection, in which this behavior is related to the membrane MWCO and to the antibiotic MW and its physicochemical properties, as discussed above. Conversely, the transmembrane pressure (factor *E*) has no significant influence (*p* = 0.220) on the rejection, but when associated with factor *A* and with the second-order interaction *AD*, resulting in the second-order interaction *AE* (−0.680) and in the third-order interaction *ADE* (−0.671), respectively, it gains greater relevance.

### 4.4. Modeling for Predictions of Permeate Flux and Antibiotic Rejection

Based on the results obtained in the 2^5^ factorial design, the regression equations for the response variables, rejection (Equation (4)), and permeate flux (Equation (5)) were achieved, considering only the factors and interactions as statistically significant. Besides, both response variables (rejection and permeate flux) were calculated using the coded values for the factors reported in Table 2—that is, with values between the levels – 1 and + 1. The non-significant factors (*p* > 0.05) were removed. Thus, the significant factors investigated for rejection were *A* (antibiotic MW) and *D* (membrane MWCO), as well as the interactions *AD*, *AE*, and *ADE*. Coefficients were defined by the ordinary least squares. Then, for rejection, the following relationship was obtained:

(4) Rejection=89.449+7.838A−9.103D+7.683AD−0.680AE−0.671ADE

For the permeate flux, except for factor *B*, all other main factors were significant, as well as the interactions *AB*, *AD*, *AE*, *CD*, *CE*, *DE*, *ABD*, *ADE*, and *CDE*, yielding the following relationship:

(5) $$\begin{array}{ll} Permeate\;flux= & 62.784-2.064A-1.369C+30.120D+25.364E-0.888AB\\ & -2.148AD-0.968AE-0.876CD-0.647CE+11.707DE\\ & -0.798ABD-1.116ADE-0.630CDE \end{array}$$

**Figure 3:**
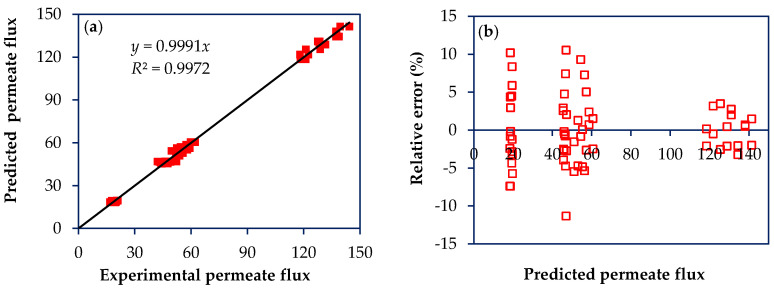
Scatter diagram for the permeate flux model. (**a**) Plot of predicted permeate fluxes vs. experimental permeate fluxes; (**b**) Plot of relative error as a function of predicted permeate flux. Permeate fluxes are given in kg h^−1^ m^−2^.

**Figure 4:**
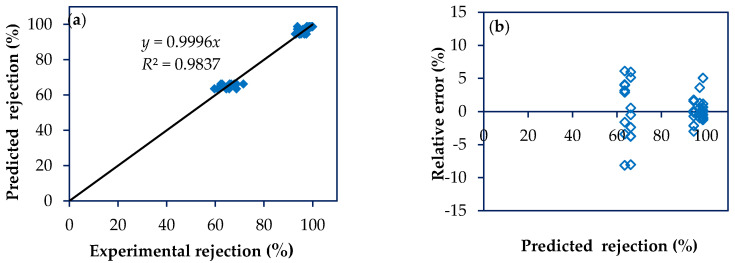
Scatter diagram for the rejection model. (**a**) Plot of predicted rejections vs. experimental rejections; (**b**) Plot of relative error as a function of predicted rejection.

Based on the experimental results of the factorial design, an internal validation of the model was performed, as reported in other work [22]. Figure 3a and Figure 4a show the scatter diagram of the permeate flux and rejection to the antibiotics by plotting experimental versus predicted values. For both response variables, the coefficients of determination (*R*^2^) and adjusted determination (*R^2^*_adj_) were greater than 0.98, showing that the model has a good agreement with experimental results obtained for NF97 and NF270 membranes treating solutions containing antibiotics with MW from 253 to 319 Da, namely SMX and NOR. Besides, low values for relative error are reported for both response variables, permeate flux and rejection, with average values of 3.41 ± 1.22% and 1.59 ± 0.86%, respectively (Figure 3b and Figure 4b).

### 4.5. External Model Validation

**Figure 5:**
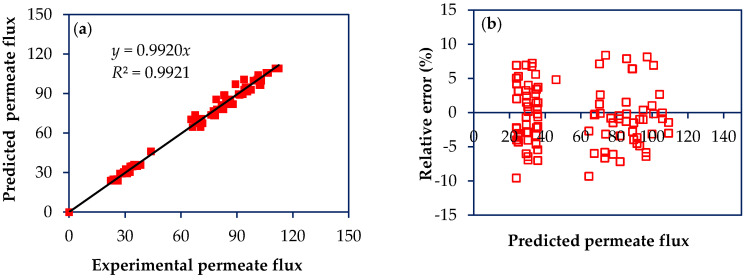
Scatter diagram for permeate flux data external to the model. (**a**) Plot of predicted permeate fluxes vs. experimental permeate fluxes; (**b**) Plot of relative error as a function of predicted permeate flux. Permeate fluxes are given in kg h^−1^ m^−2^.

**Figure 6:**
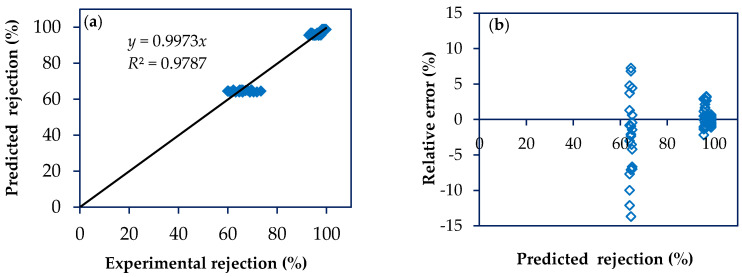
Scatter diagram for rejection data external to the model. (**a**) Plot of predicted rejections vs. experimental rejections; (**b**) Plot of relative error as a function of predicted rejection.

The model to predict permeate flux and rejection was tested with 100 additional experimental runs, that is, experiments that were not part of the model construction being considered as an external validation. Figure 5a and Figure 6a show a good fit between the experimental results and the predicted values for both response variables, permeate flux (*R*^2^ > 0.99) and rejection (*R*^2^ > 0.97), respectively. Furthermore, low relative errors were achieved for both response variables (Figure 5b and Figure 6b).

### 4.6. Effect of the Wastewater Background Matrix on the NF Performance

Table 6 shows the NF performance, regarding NOR and SMX rejection and permeate flux, as tertiary treatment of a real urban wastewater, in which the experimental results are compared with the data predicted by the model developed with synthetic solutions (Equations (4) and (5)).

The tightest membrane (NF97) presented very close rejection values, predicted and experimental ones, for both antibiotics (SMX and NOR). On the contrary, this membrane presented predicted values for permeate flux lower than the experimental ones. The loose membrane (NF270) also showed very close predicted and experimental NOR rejections, as well as lower predicted values for permeate flux than the values of the experimental results. On the other hand, NF270 presented a substantially greater rejection of SMX in the experimental results using the real wastewater, with values about 50% greater than those predicted by the model (Table 6). These results show that rejection and permeate flux were influenced by the wastewater background matrix, once it contains organic and inorganic compounds (Table 3), in which the loose membrane (NF270) is the most affected, especially regarding the rejection of the antibiotic (SMX) with lower MW. As reported in the literature [43,44], pharmaceutical compounds (e.g., antibiotics) can interact with the functional groups present on organic matter forming macromolecular complexes. In turn, these increase the effect of steric hindrance and the adsorption of pharmaceutical compounds on the membrane surface or inside its pores caused by the hydrophobicity of organic matter [10], increasing the rejection and reducing the permeate flux. In addition, the higher rejection presented by NF270 treating the urban wastewater could be associated with the inorganic background (wastewater conductivity approximately 430 µS cm^−1^), once it has already been noticed [45] that the ion adsorption may narrow the NF membrane pores. 

## 5. Conclusions

Nanofiltration was applied to the treatment of SMX- and NOR-containing wastewater, with tight and loose NF membranes and different operational conditions. The tightest membrane (NF97) presented, in all conditions investigated, the best results regarding antibiotics rejection (higher than 97%), but the lowest permeate fluxes. Conversely, the NF270 membrane showed lower rejections (65–97%) to the smallest antibiotic (MW approximately 253 Da), in which the rejection was dependent on aqueous matrix and permeate fluxes about three-fold higher than the ones achieved with the NF97 membrane. These results confirmed that the characteristics of membrane and solute, operating conditions, as well as the water background, play an important role in the nanofiltration of wastewater, as was demonstrated with the synthetic solutions and with the real wastewater studied. 

A multiple linear regression model was applied to predict the rejection and the permeate flux for both membranes and for the SMX- and NOR-containing synthetic solutions and real wastewater. A good agreement between the predicted and the experimental values was achieved, showing that this model may be applied as a tool to the scaling up of processes for the treatment of wastewater with low organic charge. Further experimental and theoretical deepening studies are important for the treatment of urban wastewater and other more complex wastewater.

## Figures and Tables

**Table 1:** Physicochemical characteristics of norfloxacin (NOR) and sulfamethoxazole (SMX).

Physicochemical Characteristic	NOR	SMX
Molecular formula ^1^	C_16_H_18_FN_3_O_3_	C_10_H_11_N_3_O_3_S
Molecular weight ^1^ (Da)	319.33	253.28
Structural formula ^1^	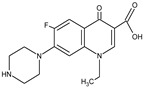	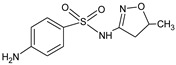
pKa ^1^	5.58; 8.68	1.97; 6.16
log K_OW_^2^	−1.03	−0.89
Polarisability ^1^ (A°)	31.15	24.16
Stokes radius ^2^ (nm)	0.47	0.39
Diffusion coefficient (10^−1^ m^2^ s^−1^)^2^	5.22	6.25

^1^ From [31]; ^2^ Obtained from the literature [32].

**Table 2:** Factors and levels investigated during the 2^5^ factorial design. MW: molecular weight, MWCO: molecular weight cut-off.

Factor	Level (−1)	Level (+1)
*A*, Antibiotic MW (Da)	253	319
*B*, Feed flow rate (L h^−1^)	480 ^1^	850 ^2^
*C*, Concentration (mg L^−1^)	5	25
*D*, Membrane MWCO (Da)	200	400
*E*, Transmembrane pressure (bar)	6	16

^1 and 2^ the respective tangential feed velocities are 0.76 and 1.34 m s^−1^.

**Table 3:** Characteristics of the secondary treated wastewater used in nanofiltration (NF) experiments.

Parameter	NF Feed Wastewater ^1^
pH	6.01 ± 0.11
Conductivity (µS cm^−1^)	428 ± 14.1
Total solids (mg L^−1^)	539 ± 139
Total suspended solids (mg L^−1^)	3.90 ± 0.60
Dissolved organic carbon – DOC (mg L^−1^)	8.50 ± 0.40
Chemical oxygen demand – COD (mg O_2_ L^−1^)	184 ± 192
NOR (mg L^−1^)	9.40 ± 3.00
SMX (mg L^−1^)	9.10 ± 1.40

^1^ Secondary treated wastewater collected + addition of 10 mg L^−1^ NOR and SMX + addition of HCl 1:1 until pH 6.0 + filtration through a 0.45 µm membrane.

**Table 4:** Effects of the MW and concentration of the antibiotic and of the feed flow rate on the permeability of NF97 and NF270 membranes.

Antibiotic	MW (Da)	Concentration (mg L^−1^)	*Q*_F_ (L h^−1^)	NF97		NF270	
				*L*_PS_ ^1^	*L*_PW_*− L*_PS_ (%)	*L*_PS_ ^1^	*L*_PW_*− L*_PS_ (%)
NOR	319	5	480	2.99 ± 0.05	3.55	8.40 ± 0.10	4.22
		5	850	3.04 ± 0.22	1.94	8.29 ± 0.23	5.47
		25	480	2.90 ± 0.13	6.45	7.91 ± 0.15	9.81
		25	850	3.01 ± 0.05	2.90	7.89 ± 0.13	10.0
SMX	253	5	480	3.01 ± 0.01	2.90	8.93 ± 0.01	−1.82
		5	850	3.20 ± 0.02	−3.23	9.25 ± 0.04	−5.47
		25	480	2.75 ± 0.08	11.3	8.58 ± 0.45	2.17
		25	850	3.04 ± 1.94	1.94	8.49 ± 0.57	3.19

^1^*L*_PS_ in kg h^−1^ m^−2^ bar^−1^.

**Table 5:** Effects of the MW and concentration of the antibiotic and of the feed flow rate on the rejection for NF97 and NF270 membranes.

Antibiotic	MW (Da)	Concentration (mg L^−1^)	*Q*_F_ (L h^−1^)	*R* (%)	
				NF97	NF270
NOR	319	5	480	99.6 ± 0.3	95.6 ± 1.0
		5	850	99.0 ± 0.4	95.2 ± 0.4
		25	480	98.7 ± 0.3	96.8 ± 0.8
		25	850	89.2 ± 3.0	95.0 ± 0.7
SMX	253	5	480	98.4 ± 0.1	65.3 ± 1.0
		5	850	98.4 ± 0.1	65.4 ± 2.0
		25	480	98.4 ± 0.1	63.3 ± 3.0
		25	850	98.6 ± 0.1	69.1 ± 1.1

**Table 6:** NF as a tertiary treatment of urban wastewater: antibiotic rejection and permeate flux.

Antibiotic	Membrane	Water Recovery (%)	Permeate Flux (kg h^−^^1^ m^−^^2^ bar^−^^1^)		Rejection (%)	
			Experimental	Predicted	Experimental	Predicted
NOR	NF97	65	12.7	19.27	98.08	98.72
		70	12.5	19.27	98.64	98.72
	NF270	65	38.5	55.88	97.06	97.22
		70	38.2	55.88	96.47	97.22
SMX	NF97	65	12.66	19.22	97.80	98.39
		70	12.45	19.22	98.01	98.39
	NF270	65	38.52	56.72	96.26	63.47
		70	38.14	56.76	97.01	63.47

## Supplementary Materials

The following are available online at https://www.mdpi.com/2077-0375/10/7/156/s1, Table S1: Full factorial design used in the NF permeation experiments, Table S2: Results of the coefficients of factors in relation to permeate flux, with *p* < 0.05 in bold. *A* = antibiotic MW (Da); *B* = feed flow rate (L h^−1^); *C* = antibiotic concentration (mg L^−1^); *D* = membrane MWCO (Da); *E* = transmembrane pressure (bar), Table S3: Results of the coefficients of factors in relation to rejection, with *p* < 0.05 in bold. *A* = antibiotic MW (Da); *B* = feed flow rate (L h^−1^); *C* = antibiotic concentration (mg L^−1^); *D* = membrane MWCO (Da); *E* = transmembrane pressure (bar).
