# Supplementary material for: Experimental Design as a Tool for Optimizing and Predicting the Nanofiltration Performance by Treating Antibiotic-Containing Wastewater
Source: Membranes (Basel). 2020 Jul 19;10(7):156. doi: 10.3390/membranes10070156 (PMC7408029; doi:10.3390/membranes10070156)
Supplement: Supplementary file 1 [file membranes-10-00156-s001.pdf]

membranes

Supplementary Data

Experimental Design as a Tool for Optimizing and Predicting the Nanofiltration Performance by Treating Antibiotic-Containing Wastewater

Dalva Inês de Souza[1], Alexandre Giacobbo[1,*], Eduardo da Silva Fernandes[2], Marco Antônio Siqueira Rodrigues[3], Maria Norberta de Pinho[4,5], Andréa Moura Bernardes[1]

[1] Post-Graduation Programme in Mining, Metallurgical and Materials Engineering, (PPGE3M), Federal University of Rio Grande do Sul (UFRGS), Av. Bento Gonçalves, n. 9500, Agronomia – Porto Alegre - RS, Brazil; dalvines@gmail.com; amb@ufrgs.br
[2] Post-Graduation Programme in Production Engineering, Federal University of Rio Grande do Sul (UFRGS), Av. Osvaldo Aranha, n. 99, Bom Fim – Porto Alegre - RS, Brazil; edu.silva.fernandes@gmail.com
[3] Post-Graduation Programme in Materials Technology and Industrial Processes, Pure Sciences and Technology Institute, Feevale University, Rodovia RS-239, n. 2755, Vila Nova – Novo Hamburgo – RS, Brazil.; marcoR@feevale.br
[4] Chemical Engineering Department, Instituto Superior Técnico, University of Lisbon, Av. Rovisco Pais, n. 1, 1049-001, Lisbon, Portugal; marianpinho@tecnico.ulisboa.pt
[5] Centre of Physics and Engineering of Advanced Materials, CeFEMA, Instituto Superior Técnico, University of Lisbon, Lisbon, Portugal
* Correspondence: alexandre_giacobbo@yahoo.com.br; Tel.: +55-51-3308-9428

Number of Tables: 3

Table S1. Full factorial design used in the NF permeation experiments.

Run	A	B	C	D	E	Run	A	B	C	D	E
1	-1	-1	-1	-1	-1	33	-1	-1	-1	-1	-1
2	+1	-1	-1	-1	-1	34	+1	-1	-1	-1	-1
3	-1	+1	-1	-1	-1	35	-1	+1	-1	-1	-1
4	+1	+1	-1	-1	-1	36	+1	+1	-1	-1	-1
5	-1	-1	+1	-1	-1	37	-1	-1	+1	-1	-1
6	+1	-1	+1	-1	-1	38	+1	-1	+1	-1	-1
7	-1	+1	+1	-1	-1	39	-1	+1	+1	-1	-1
8	+1	+1	+1	-1	-1	40	+1	+1	+1	-1	-1
9	-1	-1	-1	+1	-1	41	-1	-1	-1	+1	-1
10	+1	-1	-1	+1	-1	42	+1	-1	-1	+1	-1
11	-1	+1	-1	+1	-1	43	-1	+1	-1	+1	-1
12	+1	+1	-1	+1	-1	44	+1	+1	-1	+1	-1
13	-1	-1	+1	+1	-1	45	-1	-1	+1	+1	-1
14	+1	-1	+1	+1	-1	46	+1	-1	+1	+1	-1
15	-1	+1	+1	+1	-1	47	-1	+1	+1	+1	-1
16	+1	+1	+1	+1	-1	48	+1	+1	+1	+1	-1
17	-1	-1	-1	-1	+1	49	-1	-1	-1	-1	+1
18	+1	-1	-1	-1	+1	50	+1	-1	-1	-1	+1
19	-1	+1	-1	-1	+1	51	-1	+1	-1	-1	+1
20	+1	+1	-1	-1	+1	52	+1	+1	-1	-1	+1
21	-1	-1	+1	-1	+1	53	-1	-1	+1	-1	+1
22	+1	-1	+1	-1	+1	54	+1	-1	+1	-1	+1
23	-1	+1	+1	-1	+1	55	-1	+1	+1	-1	+1
24	+1	+1	+1	-1	+1	56	+1	+1	+1	-1	+1
25	-1	-1	-1	+1	+1	57	-1	-1	-1	+1	+1
26	+1	-1	-1	+1	+1	58	+1	-1	-1	+1	+1
27	-1	+1	-1	+1	+1	59	-1	+1	-1	+1	+1
28	+1	+1	-1	+1	+1	60	+1	+1	-1	+1	+1
29	+-1	-1	+1	+1	+1	61	-1	-1	+1	+1	+1
30	+1	-1	+1	+1	+1	62	+1	-1	+1	+1	+1
31	-1	+1	+1	+1	+1	63	-1	+1	+1	+1	+1
32	+1	+1	+1	+1	+1	64	+1	+1	+1	+1	+1

Table S2. Results of the coefficients of factors in relation to permeate flux, with p <0.05 in bold. A = antibiotic MW (Da); B = feed flow rate (L h^{-1}); C = antibiotic concentration (mg L^{-1}); D = membrane MWCO (Da); E = transmembrane pressure (bar).

Factor	Coefficient	*p*-value	Factor	Coefficient	*p*-value
**Constant**	62.784		**CD**	**-0.876**	**0.007**
**A**	2.064	**0.000**	**CE**	**-0.647**	**0.043**
B	0.625	0.051	**DE**	**11.707**	**0.000**
**C**	**-1.369**	**0.000**	ABC	0.144	0.645
**D**	**30.120**	**0.000**	**ABD**	**-0.798**	**0.014**
**E**	**25.364**	**0.000**	ABE	-0.429	0.174
**AB**	**-0.888**	**0.007**	ACD	-0.138	0.659
AC	-0.075	0.810	ACE	-0.121	0.698
**AD**	**-2.148**	**0.000**	**ADE**	**-1.116**	**0.001**
**AE**	**-0.968**	**0.003**	BCD	0.155	0.620
BC	0.428	0.175	BCE	0.529	0.096
BD	0.208	0.506	BDE	0.138	0.658
BE	0.157	0.615	**CDE**	**-0.630**	**0.049**

$R^2 = 0.9979$; $R^2_{adj} = 0.9956$.

Table S3. Results of the coefficients of factors in relation to rejection, with p <0.05 in bold. A = antibiotic MW (Da); B = feed flow rate (L h^{-1}); C = antibiotic concentration (mg L^{-1}); D = membrane MWCO (Da); E = transmembrane pressure (bar).

Factor	Coefficient	*p*-value	Factor	Coefficient	*p*-value
**Constant**	**89.449**		CD	-0.193	0.447
**A**	**7.838**	**0.000**	CE	**0.117**	0.645
B	-0.201	0.429	DE	0.247	0.332
C	0.220	0.386	ABC	-0.092	0.716
**D**	**-9.103**	**0.000**	ABD	-0.065	0.797
E	0.313	0.220	ABE	0.134	0.597
AB	-0.420	0.102	ACD	-0.002	0.993
AC	0.290	0.255	ACE	-0.246	0.334
**AD**	**7.683**	**0.000**	**ADE**	**-0.671**	**0.011**
**AE**	**-0.680**	**0.010**	BCD	-0.027	0.914
BC	0.138	0.586	BCE	-0.351	0.171
BD	0.130	0.608	BDE	0.027	0.914

$R^2 = 0.9884$; $R^2_{adj} = 0.9808$.

© 2020 by the authors. Submitted for possible open access publication under the terms and conditions of the Creative Commons Attribution (CC BY) license (http://creativecommons.org/licenses/by/4.0/).
